# Neuronal Basis of Innate Olfactory Attraction to Ethanol in *Drosophila*

**DOI:** 10.1371/journal.pone.0052007

**Published:** 2012-12-20

**Authors:** Andrea Schneider, Manuela Ruppert, Oliver Hendrich, Thomas Giang, Maite Ogueta, Stefanie Hampel, Marvin Vollbach, Ansgar Büschges, Henrike Scholz

**Affiliations:** 1 University of Cologne, Biocenter, Zoological Institute, Department of Animal Physiology/Neurobiology, Cologne, Germany; 2 Instituto de Neurociencias de Castilla y León, Departmento Biología Celular y Patología, Salamanca, Spain; 3 Howard Hughes Medical Institute, Janelia Farm Research Campus, Ashburn, Virginia, United States of America; University of Missouri, United States of America

## Abstract

The decision to move towards a mating partner or a food source is essential for life. The mechanisms underlying these behaviors are not well understood. Here, we investigated the role of octopamine – the invertebrate analogue of noradrenaline – in innate olfactory attraction to ethanol. We confirmed that preference is caused via an olfactory stimulus by dissecting the function of the olfactory co-receptor Orco (formally known as OR83b). Orco function is not required for ethanol recognition per se, however it plays a role in context dependent recognition of ethanol. Odor-evoked ethanol preference requires the function of Tbh (Tyramine β hydroxalyse), the rate-limiting enzyme of octopamine synthesis. In addition, neuronal activity in a subset of octopaminergic neurons is necessary for olfactory ethanol preference. Notably, a specific neuronal activation pattern of tyraminergic/octopaminergic neurons elicit preference and is therefore sufficient to induce preference. In contrast, dopamine dependent increase in locomotor activity is not sufficient for olfactory ethanol preference. Consistent with the role of noradrenaline in mammalian drug induced rewards, we provide evidence that in adult *Drosophila* the octopaminergic neurotransmitter functions as a reinforcer and that the molecular dissection of the innate attraction to ethanol uncovers the basic properties of a response selection system.

## Introduction

Preference is a fundamental behavior that determines whether an animal approaches a food source or not. Ethanol preference guides the fruit fly *Drosophila melanogaster* to a fermenting fruit, a putative food source and/or oviposition site [1]–[3]. The behavior might be influenced by internal factors such as ethanol metabolism, since impaired Alcohol dehydrogenase activity, important for the degradation of ethanol, correlates with loss of preference for low ethanol concentrations [1]. In addition to internal factors external factors are required to induce preference behavior. For example ethanol emanated from a fermenting food source attracts animals like butterflies over long distance [4]. Therefore, ethanol preference can be viewed as targeted approach behavior based on an olfactory cue. However, the knowledge on how this behavior is generated on the neuronal level is far from being comprehensive.

To elicit this approach behavior complex information processing has to occur at different anatomical levels in the brain. First the odor has to be perceived. That includes odor recognition and discrimination. In *Drosophila* odor perception occurs on the level of olfactory neurons which express odor specific receptors and the broadly expressed general co-receptor Orco [5]; [6]. After activation of the olfactory neurons the signal is processed in the antennal lobes, a structure involved in transmission and habituation of olfactory information to the projection neurons [7]. The projection neurons in turn project to the lateral horn and the mushroom bodies, structures involved in associative olfactory learning and memory [8]; [9]. Positive and negative short-term memories of an olfactory stimulus require the output of the mushroom bodies [10]. Innate olfactory attraction can be initiated on the level of the antennal lobes. Activation of particular olfactory glomeruli in response to a natural odor causes approach behavior, whereas activation of other glomeruli can cause aversion [11]. The decision to react to an odor stimulus with approach behavior has to be coordinated with the multiple locomotor system networks involved in the decision to move, the determination of movement characteristics and movement execution [12].

The biogenic amine octopamine has been implicated in different levels of odor processing in insects [13]. For example, octopamine increases the firing rate in olfactory receptors neurons in response to pheromone stimulation in the silk moth *Bombyx mori* [14]. In the honey bee *Apis mellifera* octopamine receptors are expressed in the lateral interneurons of the antennal lobes suggesting they might be involved in modulating olfactory information processing [15]. Furthermore, in honey bees octopamine acts as a positive reinforcer in olfactory learning within the olfactory pathway [16], [17]. In *Drosophila* octopaminergic neurons are required in appetitive odor evoked short term memory formation whereas dopaminergic neurons are required for aversive memory [10].

The role of octopamine signaling in adult olfactory ethanol preference has not been analyzed. In addition, it is not known whether octopamine plays an instructive role in the adult fly. It is further not clear whether dopamine-dependent changes in locomotor activity are involved in olfactory ethanol preference. Here, we show that Orco functions in odor dependent discrimination, a function required for olfactory ethanol preference. Olfactory ethanol preference requires TbH activity in a subset of octopaminergic neurons. Activation of a subset of TbH/octopaminergic neurons is both necessary and sufficient to induce preference. In addition, increased locomotor activity cannot account for olfactory preference.

## Results

### Odor Evokes Ethanol Preference

**Figure 1:**
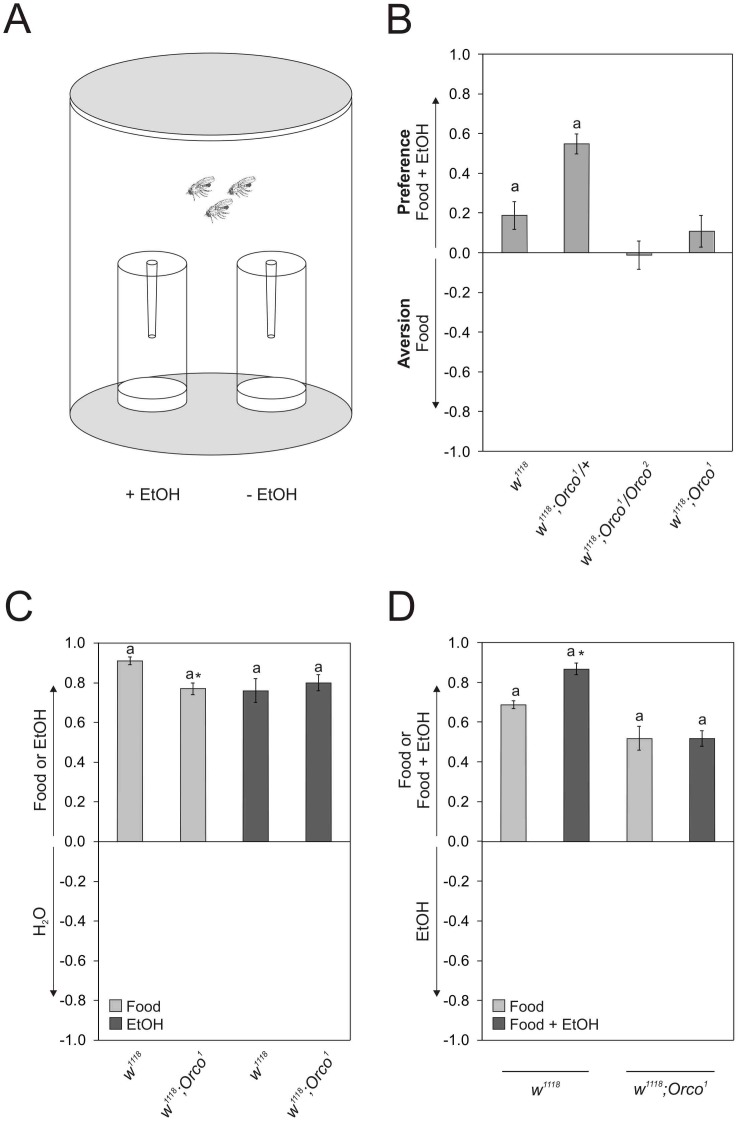
Ethanol preference is based on olfactory information. a Flies are offered a choice between two odor traps and flies will normally decided for one or the other trap within 16 h. The preference index (PI) indicates the percentage of flies that prefer one odor over the other. A positive PI is defined as preference and a negative PI indicates aversion. **b** Control and heterozygote *Orco^1^* mutants prefer ethanol containing food odors, whereas transheterozygote *Orco^1^/Orco^2^* and homozygote *Orco^1^* mutants do not show preference (PIs are *w^1118^* 0.19±0.07, *Orco^1^/+*0.55±0.05, *Orco^1^/Orco^2^* −0.01±0.07 and *Orco^1^*/*Orco^1^* 0.11±0.08. n = 24, 26, 32 and 31. *P*<0.01 and *P*<0.001). **c** Flies prefer food odors over water (PIs are *w^1118^* 0.91±0.02 and *w^1118^*; *Orco^1^* 0.77±0.03. n = 29. There is a significant difference for food preference between *w^1118^* and *Orco^1^*. *P*<0.05) and ethanol over water (PIs are *w^1118^* 0.76±0.06 and *w^1118^*;*Orco^1^* 0.8±0.04. n 11 and 13.). **d** Grey bars represent the choice between food odor and ethanol (PIs are *w^1118^* 0.69±0.04 and *w^1118^*;*Orco^1^* 0.52±0.05. n 33.) and dark grey indicates the choice between food odor with ethanol versus ethanol alone (PI are w^1118^ 0.87±0.03 and *w^1118^*;*Orco^1^* 0.52±0.06. n = 29 and 30. The olfactory preferences of *w^1118^* for different tested conditions differ significantly. *P*<0.05). Bars labeled with **a** are significantly different from random choice as determined by One-sample sign test.

When offered a choice between food odors containing ethanol and food odors without ethanol in a two odor choice paradigm *Drosophila melanogaster* prefers the ethanol enriched food source ([1]; Figure 1a, 1b). To determine whether the odor trap assay indeed measures olfactory ethanol preference we analyzed flies with impaired odor perception. In *Drosophila* olfactory co-receptor Orco is essential for olfaction [5]. Mutants lacking Orco function are, for example, impaired in the perception of the natural occurring smell of vinegar [11]. Consistent with the idea that *Orco* mutants are impaired in the sensory perception of natural odors, homozygote *Orco^1^* mutants and transheterozygote *Orco^1^*/*Orco^2^* mutants did not show preference for ethanol-containing food odors (Figure 1b). To test whether they are indeed anosmic for ethanol and/or food odors, *Orco* mutants were given a choice between ethanol or food odor and water (Figure 1c). The *Orco^1^* mutants preferred ethanol and food odors over water. *Orco^1^* mutants were significantly less attracted to food odors than control flies suggesting they might have a reduced sensibility for those odors. The *Orco^1^* mutants detect the smell of ethanol and food and thus can perceive odors. In general, flies prefer complex odor mixtures over single odors and therefore can distinguish between different kinds of odors [2]. To test whether *Orco* mutants can distinguish between a complex odor mixture (food odor) and a single odor (ethanol), *Orco^1^* mutants were offered a choice between ethanol and food odors (Figure 1d). Control and *Orco^1^* mutant flies preferred complex food odors over single odors, consistent with the fact that the flies can distinguish between complex and single odors. However, *Orco^1^* mutants preferred food odors to a lesser extent supporting the notion that they might be less sensitive to those odors.

About 69% of the control flies preferred food over ethanol odor as reflected by a preference index (PI) of 0.69±0.04. However, around 31% of the flies still decided to enter the odor trap with ethanol. To shift the weight of attractiveness between the presented odors we addressed whether adding ethanol to a complex odor makes it even more attractive than the single ethanol odor. Indeed, for control flies adding ethanol to a complex food odor significantly increased their preference (Figure 1d). Now approx. 87% of the flies prefer the food odor over pure ethanol. Interestingly, *Orco^1^* mutants did not increase their preference. Taken together with the observation that *Orco^1^* mutants can sense and distinguishe between ethanol and food odors, but did not distinguish between food odor with and without ethanol this result suggests that *Orco^1^* mutants fail to recognize changes in the complexity of the odor environment. Therefore, we conclude that Orco functions in context dependent recognition of the ethanol vapor. Our data are consistent with the fact that the assay measures olfactory ethanol preference.

### TβH is Required for Olfactory Ethanol Preference

**Figure 2:**
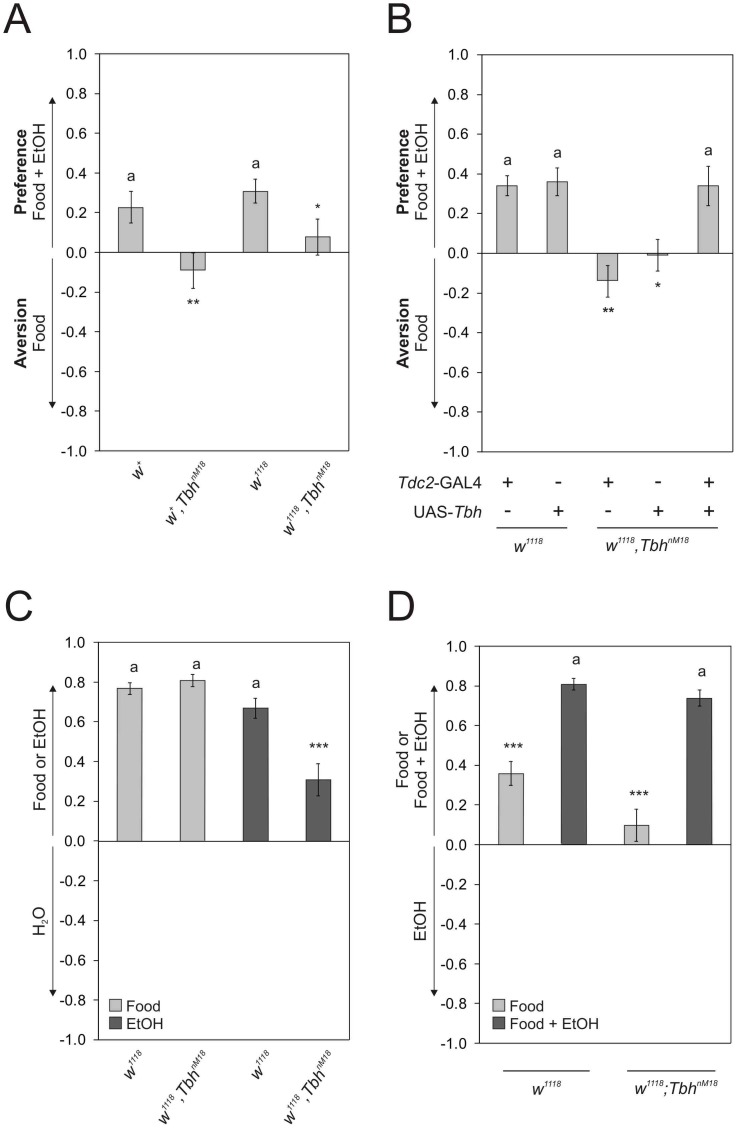
Ethanol preference depends on TbH function. **a** *Tbh^nM18^* mutants do not show preference and differ significantly from controls (PIs are *Tbh^nM18^* 0.09±0.09 and control 0.23±0.08. n 21 and 23. *P*<0.01). The loss of preference is independent of *w^1118^* (PIs are *w^1118^* 0.31±0.06 and *w^1118^*, *Tbh^nM18^* 0.08±0.09. n 26 and 17. *P*<0.05 and *P*<0.01). **b** *Tdc2*-GAL4 dependent expression of TbH in *Tbh^nM18^* mutants restores preference to control levels (PIs are *w^1118^*;*Tdc2*-GAL4/+0.34±0.05, *w^1118^*; UAS-*TbH* 0.36±0.07, *w^1118^*,*Tbh^nM18^*;*Tdc2*-GAL4/+ −0.14±0.08, *w^1118^*,*Tbh^nM18^*;UAS-*TbH*/+ −0.01±0.08 and *w^1118^*,*Tbh^nM18^*;*Tdc2*-GAL4/UAS-*TbH* 0.34±0.1. n 21, 28, 23, 21 and 21.). Mutants carrying the transgenes insertions in the mutant background differ significantly from control carrying the respective transgene (*P*<0.05 and *P*<0.01). **c** *Tbh^nM18^* mutants prefer food odor over water similar to the controls (PIs are for *w^1118^* 0.77±0.03 and *w^1118^*, *Tbh^nM18^* 0.81±0.03. n 18 and 15.) but do not prefer ethanol odor over water to the same extend as the controls (PIs are *w^1118^* 0.67±0.05 and *w^1118^*,*Tbh^nM18^* 0.31±0.08. n 20 and 21. *P*<0.001). **d** Control flies prefer complex odors over the single odor ethanol (PI is *w^1118^* 0.36±0.06. n 35.) and increase their preference significantly for the complex odor when ethanol is added (PI is *w^1118^* 0.81±0.03. n 18, *P*<0.001). *Tbh^nM18^* flies show an increased preference for complex odors with ethanol over ethanol in comparison to the choice of complex odors without ethanol and ethanol (*w^1118^*,*Tbh^nM18^* 0.10±0.08 and 0.74±0.04. n 24 and n 17. *P*<0.001). The letter **a** marks differences from random choice as determined by a One-sample sign test.

The positive association of an external odor stimulus depends on octopaminergic signaling [10]. To determine whether octopamine plays a role in olfactory ethanol preference, we tested *Tbh^nM18^* mutants lacking detectable amounts of octopamine for olfactory ethanol preference (Figure 2). The *Tbh^nM18^* mutants did not show olfactory ethanol preference (Figure 2a). This phenotype, however, did not depend on the *white^1118^* mutation, which was used as the genetic background of the experiments. To confirm that the loss of preference was due to the loss of TbH function, we restored this function in tyraminergic/octopaminergic neurons by using a GAL4 construct under control of the *Tdc2* (*Tyrosine decarboxylase 2*) promotor [18]. Indeed, preference can be restored by expressing a *Tbh* transgene under the control of the *Tdc2*-GAL4 driver line. The transposable element insertions of the used transgenes did not influence the loss of olfactory ethanol preference of *Tbh^nM18^* mutants (Figure 2b). Therefore, TbH is required for olfactory ethanol preference.

One requirement for olfactory preference is the ability to recognize ethanol or food odors. Control and *Tbh^nM18^* flies preferred food odors over water (Figure 2c). Although *Tbh^nM18^* mutants showed preference for ethanol over water this preference was reduced, suggesting that they might be less sensitive to ethanol (Figure 2c). However, increasing ethanol concentrations when food odors and food odors with ethanol are offered, did not lead to olfactory ethanol preference either in *Tbh^nM18^* mutants (Table S1). In addition, *Tbh^nM18^* mutants distinguished between a single odor and a complex food odor with or without ethanol (Figure 2d). Hence, the lack of preference for ethanol containing food odors was not due to an impaired odor perception including a reduced sensitivity to ethanol or a failure to discriminate complex and single odors in *Tbh^nM18^* mutants.

To identify TbH – and in turn octopamine – positive neurons involved in olfactory ethanol preference, we first looked at the TbH expression pattern in the adult brain and thorax using an antibody raised against TbH [19]. Expression of TbH was found in around 112 neurons in the adult brain and in about 39 in the ventral nerve cord (VNC) (Table S2, S3, Figure S4). We compared the number and location of TbH expressing cells with previously described octopaminergic neurons [20]–[23]. In the G0b, G3a/AL2, VMI-VMIII clusters the number of TbH expressing soma and octopamine positive neurons matched. However, we also found clusters that had more TbH positive neurons (the G2b and G4a clusters) or less TbH positive cells (the G3b and G0 clusters). We also identified a neuron that has not been previously described posterior to the described G0a cluster and that we therefore named G0 posterior. In the VNC around 18 octopamine positive neurons have been identified so far [20]. Here, we identified a total of around 39 TbH positive neurons (Table S3, Figure S4). In general, we find TbH expressing clusters in similar regions as previously described octopaminergic cells. However, on average the clusters have more TbH positive neurons. The reduced number of octopamine positive neurons in comparison to TbH expressing neurons might be due to differences in sub-cellular localization and/or turnover rate of TbH and octopamine. In a subpopulation of tyraminergic/octopaminergic neurons the octopamine concentration can change dynamically due to the previous experience of the animal [24].

**Figure 3:**
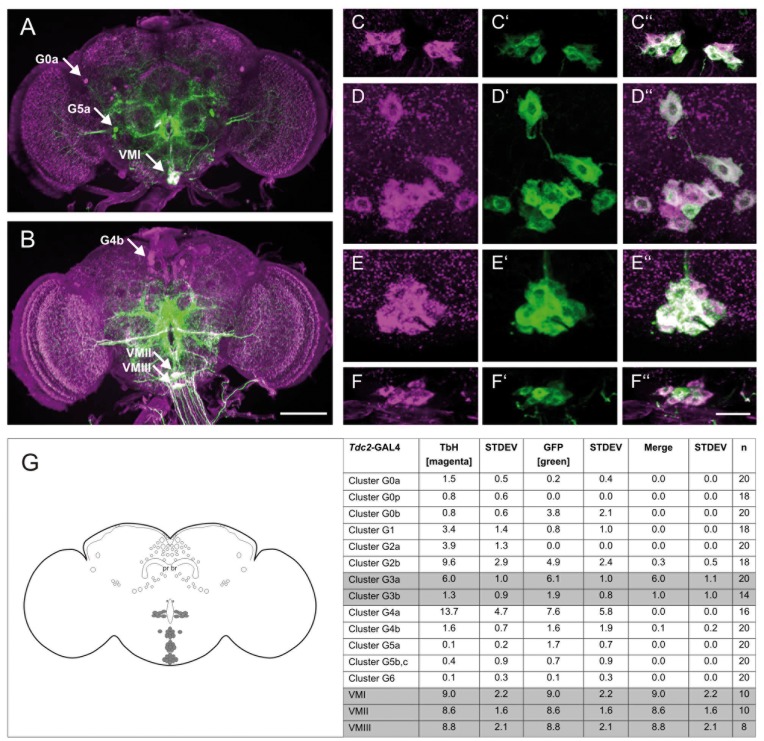
The *Tdc2*-GAL4 activates *UAS* transgenes in a subset of TbH positive neurons. The GAL4 expression domain of the *Tdc2*-GAL4line is visualized by GFP in comparison to TbH expression in the anterior (**a**) and posterior (**b**) part of the adult brain. Neurons expressing TbH (G0a) or GFP (G5a) and neurons expressing both (VMI) are highlighted with an arrow (**a**). In the back of the brain G4a neurons only express TbH, whereas VMII and VMIII neurons express both (**b**). The expression of Tbh (**c** to **f**) and GFP (**c′** to **f′**) is found in the same set of neurons of the G3a/AL2 cluster (**c′′**), VMI (**d′′**), VMII (**e′′**) and VMIII (**f′**). The analysis of expression patterns is summarized in table **g** and clusters in which GFP and Tbh co-localize are highlighted in grey. The scale bar represents 25 µm.

To determine in which subset of neurons TbH is required for olfactory ethanol preference, we compared the expression pattern of the *Tdc2*-GAL4 driver line visualized with a UAS-*mCD8::GFP* transgene [25] with the expression of TbH (Figure 3, Table S2 and S3). In the adult brain, some cells express only Tbh (the G0a and G4b clusters) or GFP (the G5a cluster) (Figure 3a and 3b). TbH and GFP are co-expressed in the G3a/AL2 cluster, the G3b cluster, and the three ventral clusters (VMI to VMIII; Figure 3c to f), in which all TbH positive cells express GFP and vice versa (Figure 3g). In the VNC around 37 neurons of the 39 TbH expressing neurons also express GFP (Table S2 and S3). If TDC2 is indeed required for tyramine and octopamine synthesis and there are no alternative pathways involved in the synthesis of octopamine, the employed promoter fragment of the *Tdc2*-GAL4 line does not reflect the comprehensive expression profile of endogenous TDC2. Consistently with this assumption, octopamine positive neurons have been identified that are not targeted by the *Tdc2*-GAL4 [23]. Taken together, in the adult fly the *Tdc2*-GAL4 lines targets around 78 TbH expressing neurons in the brain and VNC. The function of TbH within neurons of this subset is g003required for olfactory ethanol preference.

### A Subset of Octopaminergic Neurons is Involved in Olfactory Preference

**Figure 4:**
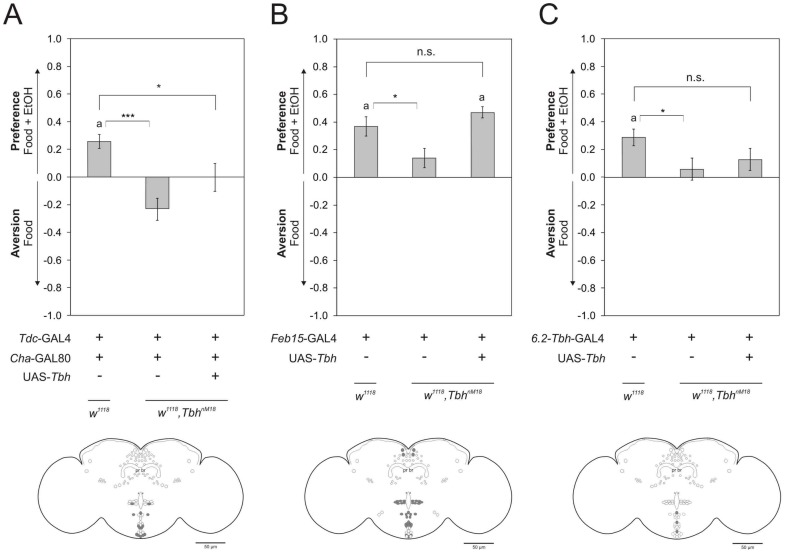
TbH is required in a subset of octopamingergic neurons for ethanol preference. **a** Expression of TbH in a *Tdc2*-GAL4; *Cha*-GAL80 dependent manner in *Tbh^nM18^* mutants does not restore preference (PIs are *Tdc2*-GAL4/+; *Cha*-GAL80/+: 0.26±0.05, *Tbh^nM18^*; *Tdc2*-GAL4; *Cha*-GAL80/+0.23±0.08 and *Tbh^nM18^, UAS-Tbh*; *Tdc2*-GAL4; *Cha*-GAL80/+0.00±0.10, n 27, 22 and 25. The experimental group does not develop preference and differs significantly from the control. The transgene insertion in the mutant background differs significantly from respective control. *P*<0.05 and <0.001.). **b** *Feb15*-GAL4 dependent expression of TbH in *Tbh^nM18^* mutants restores preference (PIs are *w^1118^; Feb15*-GAL4/+0.37±0.07; *w^1118^*, *Tbh^nM18^; Feb15*-GAL4/+0.14±0.07 and *w^1118^, Tbh^nM18^*, *UAS-Tbh*; *Feb15*-GAL4/+0,47±0.04. n 28, 33 and 36. The level of preference of the experimental group does not differ from the control. The transgene insertion in the mutant background differs significantly from respective control. *P*<0.05.). **c** Loss of preference in *Tbh^nM18^* mutants is not completely restored by TbH expression under the control of the 6.2-T*bh*-GAL4 driver (PIs are *w^1118^; 6.2-Tbh*-GAL4/+0.29±0.06, *w^1118^, Tbh^nM18^; 6.2-Tbh-*GAL4/+0.06±0.08 and *w^1118^, Tbh^nM18^*, *UAS-Tbh*; *6.2-Tbh*-GAL4/+0.13±0.08. n 36, 26 and 36. The olfactory preferences of the control group and experimental group are not significantly different. Mutants carrying one copy of the GAL4 transgene differ significantly from the control carrying one copy of the GAL4 transgene. *P*<0.05.). The schemata below graphs for behavioral experiments summarize the expression domains of respective GAL4 drivers in comparison to TbH expression. White cells are TbH positive neurons not targeted by specific driver lines and grey cells indicate co-localization of GAL4 and TbH. The letter **a** labels an olfactory preference score that is significantly different from random choice as determined by *One-sample* sign test.

The GAL4 expression domain of the *Tdc2*-GAL4 driver can be further restricted by the expression of a *Cha*-GAL80 transgene. This subset of octopaminergic neurons is involved in mediating aggression [19]. *Cha*-GAL80 contains a *Cha* (*Choline acetyltransferase*) promotor fragment and suppresses the expression of GAL4 in cholinergic neurons [26]. To address whether the failure of *Tbh^nM18^* mutants to prefer ethanol is due to the inability to move towards an identified object, we expressed TbH under the control of *Tdc2*-GAL4 and *Cha*-GAL80 in *Tbh^nM18^* mutants (Figure 4a). The expression of TbH in those flies fails to rescue the loss of olfactory ethanol preference. Thus, the TbH expressing neurons mediating preference differ from the subset involved in aggressive behaviors.

To determine which TbH expressing neurons are involved in mediating preference, we compared the GAL4 expression patterns of *Tdc2*-GAL4, *Cha*-GAL80 and *Tdc2*-GAL4 with respect to the TbH expression pattern (Figure 4a; Figure S1, S5 and Table S3). In the brain the *Tdc2*-GAL4, *Cha*-GAL80 line expresses GAL4 in 14 out of 112 TbH neurons including cells in the G3a/AL2, the G3b, the VMI-III cluster, and in 32 out of 39 TbH expressing neurons in the VNC (Table S3 and Figure S5). From comparison, we could exclude the TbH positive cells of the G3b cluster in the brain and all in the VNC except the aDUM from being involved in mediating preference. Hence, mediation of ethanol preference resides in the brain in a subset of G3a/AL and VMI to VMIII neurons and/or one of the VNC’s dorsal unpaired median neurons (aDUM).

To independently confirm that cells of the VM and G3a/AL2 are involved in mediating preference we used the *Feb15*-GAL4 driver for rescue experiments (Figure 4b). The expression of TbH under the control of the *Feb15*-GAL4 driver in *Tbh^nM18^* mutants restores preference. The *Feb15*-GAL4 driver expresses GAL4 in all neurons of the G3a, VMI-VMII clusters, and in a subset of cells of the VMIII cluster (Figure 4b, Figure S2). There is no expression of GAL4 in the aDUM (Table S3). This is consistent with the idea that neurons of the G3a and VMI-III clusters are involved in mediating preference. However not all neurons are required.

To independently confirm that the G3a and VM clusters are required for olfactory ethanol preference, we used the GAL4 driver *NP7088* to restore TbH expression in *Tbh^nM18^* mutants (Figure S6). The expression of TbH under the control of the *NP7088*-GAL4 driver in *Tbh^nM18^* mutants restores olfactory ethanol preference. The GAL4 expression pattern of the *NP7088*-GAL4 and the *Tdc2*-GAL4 line overlap only in octopaminergic neurons of the G3a and VM clusters (Busch et al., 2009). In comparison to the expression domain of the *Feb15*-GAL4 line, no GFP expression was found in the G3b and G4b clusters ruling out that over-expression of TbH in these clusters using the *Feb15*-GAL4 driver restore the loss of preference in *Tbh^nM18^* mutants. The results are consistent with the conclusion that expression of TbH in the G3a and VM clusters is required for olfactory ethanol preference.

To address whether a TbH positive ventral unpaired median neuron (VUM) with VUMa4 morphology is required for ethanol preference, the *6.2-Tbh-*GAL4 driver was used to restore preferences in *Tbh^nM18^* mutants (Figure 4c, Figure S3, Table S3). Expression of TbH in this neuron in TbH deficient flies did not restore preference. However it decreased the difference between the control and the experimental group, suggesting that TbH in this neuron partially rescues preference. Thus, it is unlikely that TbH in the VUMa4 neuron is required for olfactory ethanol preference alone.

In summary 26 TbH expressing neurons of the G3a/AL2 cluster and the VMI-VMIII are involved in ethanol preference. The comparison of the different GAL4 expression in the thoracic ganglion revealed that Tbh positive neurons in the VNC do not mediate preference. Therefore, Tbh dependent generation of movements at the level of the VNC is not required for ethanol preference. Comparative studies with octopamine and the expression domain of the*Tdc2*-GAL4 driver showed that all these neurons also express octopamine [23]. Therefore, we conclude that ethanol preference is mediated by an octopaminergic subpopulation of neurons in the G3a/AL2 and VMI-VMIII cluster.

### Neuronal Activity is Necessary and Sufficient to Induce Preference

**Figure 5:**
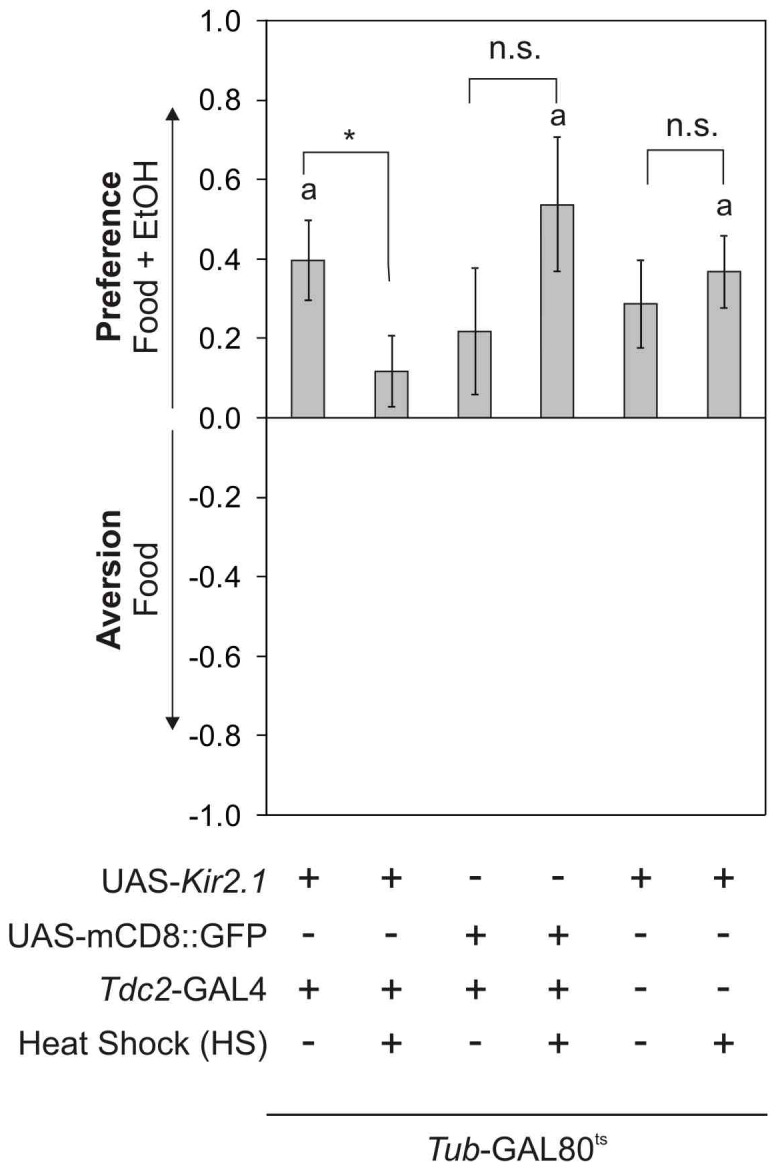
Neuronal activity is required for preference. After activation of UAS-Kir2.1 under the control of *Tdc2*-GAL4, *tub*-GAL80^ts^ preference is significantly reduced (PIs are *Tdc2*-GAL4, *tub*-GAL80^ts^; UAS-Kir2.1 0.4±0.1 and with HS 0.12±0.09. n 22. P<0.05). Activation of a *UAS-*mCD8::GFP construct does not interfere with preference (PIs are *Tdc2*-GAL4, *tub*-GAL80^ts^; *UAS*-mCD8::GFP 0.22±0.16 and with HS 0.54±0.17. n 14.). Transgene insertions do not alter preference of flies carrying *UAS*-Kir2.2 and *tub*-GAL80^ts^ (PIs are w^1118^; *tub*-GAL80^ts^; *UAS*-Kir2.1 0.29±0.11 and with HS 0.37±0.09. n 27 and 28.).

To determine whether neuronal activity of the octopaminergic/tyraminergic neurons is required for olfactory ethanol preference, flies with silenced neuronal activity in a *Tdc2*-GAL4 dependent manner were tested for olfactory ethanol preference. To prevent neurons from depolarization, a *KCNJ2* (*Potassium channel, inwardly rectifying, subfamily J member; also known as Kir2.1)* UAS transgene (UAS-*Kir2.1*) under the control of the *Tdc2*-GAL4 combined with a temperature sensitive GAL80 inhibitory construct was used [27], [28]. Expression of UAS-*Kir2.1* was inhibited during development by ubiquitous expression of the temperature sensitive GAL80 inhibitor. After transfer to 33°C, GAL80 dissociates from the UAS binding site and GAL4 expressed in a *Tdc2*-GAL4 dependent manner binds to the UAS sequence and activates transgene expression (Figure 5). Flies with *Tdc2*-GAL4 silenced neuronal activity failed to show preference. To show that this phenotype was due to a specific inactivation of neuronal activity and not due to unspecific interference in these neurons, we induced the expression of a UAS-*mCD8::GFP* transgene in a comparable manner. The expression of GFP did not interfere with preference. In addition, leaky expression of the transgenes does not account for a loss of preference. The results show that neuronal activity in the adult fly in octopaminergic/TbH positive neurons is required for olfactory preference.

**Figure 6:**
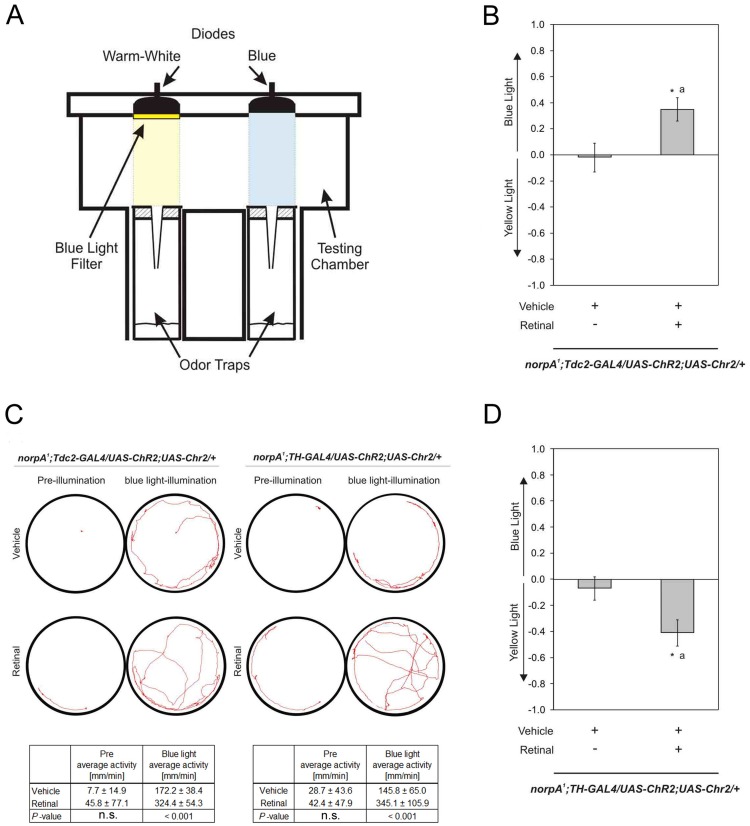
Neuronal activity is sufficient to induce preference. a The behavioral set up of the optogentic trap assay consists of two odor traps filled with food odor surrounded by a dark intransparent plastic. The assay is placed on a cold light plate to motivate flies to descend into the fly traps. On top of the two odor traps two different diodes – one for blue light and one for yellow – are placed that can be activated with the different frequencies. **b** Light activation of neurons in a *Tdc2*-GAL4 dependent manner causes preference for the trap illuminated in blue (PIs are *norp^A1^*, *Tdc2*-GAL4/*UAS*-ChR2;*UAS*-ChR2 with vehicle −0.02±0.11 and with retinal 0.35±0.09; n 12 for both. *P*<0.05.). **c** Typical traces of *norp^A1^*,*Tdc2*-GAL4/*UAS*-ChR2;*UAS*-ChR2 and *norp^A1^*,*TH*-GAL4/*UAS*-ChR2;*UAS*-ChR2 flies before and after a one min illumination period are shown. The average activity of 7 flies per genotype and condition are summarized in the table. **d** Activation of dopaminergic neurons causes aversion for the trap illuminated in blue (PIs are *norp^A1^*,*TH*-GAL4/*UAS*-ChR2;*UAS*-ChR2 with vehicle −0.07±0.09 and with retinal −0.41±0.1. n 20 and 35. *P*<0.05.).

To determine whether neuronal activity in octopaminergic/tyraminergic neurons is sufficient to induce preference, neuronal activity was induced by light activation of a UAS-*ChR2* (*Channelrhodopsin-2*) transgene under the control of the *Tdc2*-GAL4 driver (Figure 6). In the presence of all-trans retinal CHR2 is transformed into a depolarizing blue light-gated cation selective ion channel which activates neurons [29]. Experimental flies were fed with all-trans retinal whereas control flies were fed with the vehicle only. During the experiment flies were offered a choice between two odor traps filled with the same food odor. One vial was illuminated with the activating blue light and the second vial with yellow light of similar intensity and frequency (Figure 6a). Previously, it has been shown in honey bees that unconditioned stimuli in reward learning are mediated by neuronal activity of the octopaminergic VUMmx1 neuron firing with a brief frequency of around 40 Hz followed by a period of enhanced activity around 8 Hz [16]. Therefore, we decided to use a similar pattern for induction of *Tdc2*-GAL4 by blue light activation of UAS-*ChR2.* To rule out that flies respond to an optical stimulus, the *norpA^1^* mutation was introduced into the background of these flies. *norpA* (*no receptor potential A*) deficient flies are unable to generate receptor potentials in response to light and are therefore blind [30]. Control *norpA^1^* flies not activated with light chose randomly between both vials. However, *norpA^1^* flies in which *Tdc2*-GAL4 neurons were activated with the structured activity pattern preferred the vial illuminated in blue light over yellow light (Figure 6b).

We observed that activation of octopaminergic neurons by light caused an increase of locomotor activity (Figure 6c). To investigate whether induced hyperactivity would increase the likelihood to choose one vial over the other, we tested hyperactive flies in the same behavioral choice paradigm. TH-GAL4 (Tyrosine hydroxylase) dependent activation of dopaminergic neurons by light-activated ionotropic P2rx2 (purinergic receptor P2X, ligand-gated ion channel) or heat-activated TrpA1 (Transient receptor potential A1 ion channel) increases locomotor activity in slow moving flies [31], [32]. Therefore, we tested flies expressing CHR2 under the control of the TH-GAL4 driver for light induced preference (Figure 6d). The dopaminergic neurons were activated by blue light in the presence of all-trans retinal. A sample trace shows that our testing conditions indeed induce an increase in locomotion (Figure 6c). The flies showed a significant preference for yellow light and aversion to blue light. Activation of the dopaminergic neurons with the 50 Hz/5 Hz pattern noted above caused hyperactivity. We conclude that the activation of octopaminergic/tyraminergic neurons is sufficient for preference and is not caused by hyperactivity.

## Discussion

At least two components influence olfactory ethanol preference. Preference is caused by external odor stimulus as mutants lacking Orco failed to show preference. Consistently, silencing of specific glomeruli – the target area of olfactory neurons expressing Orco – also leads to loss of olfactory attraction to the natural odor vinegar [11]. The second component is uncovered by the phenotypic analysis of the *Tbh^nM18^* mutant. Olfactory ethanol preference in *Tbh^nM18^* mutants was similarly impaired as in *Orco* mutants. However, direct changes in the primary odor perception cannot account for this observed behavior. The loss of preference in *Tbh^nM18^* flies was not due to reduced sensitivity to olfactory stimulus because higher concentrations do neither cause preference. In addition, unlike *Orco* mutants, the ability to discriminate complex food odors appeared not to be impaired in *Tbh^nM18^* mutants. Consistently, in honey bees inhibition of octopaminergic signaling by blocking octopamine receptor function does not interfere with odor discrimination [33]. Therefore, Tbh does not act on the same level as Orco in mediating preference.

Lack of olfactory ethanol preference could also have resulted from changes in the execution of motor tasks associated with preference. However, this can be ruled out for several reasons. Firstly, TbH in the VNC, a region involved in the generation of locomotor output, was not required for preference. Secondly, increased locomotion was not involved in preference, as activation of dopaminergic neurons causing increases in locomotion did not result in a similar behavior. Thirdly, *Tbh^nM18^* flies appeared normal for motor tasks required in other behavioral paradigms. *Tbh^nM18^* mutants can perform motor tasks associated with an odor evoked startle response [34], flight related wing beats [35] and aggression related locomotor behaviors [19]. Taken together, these observations indicate that *Tbh^nM18^* mutants sense environmental changes and are able to perform motor related tasks, but are unable to respond to the environmental stimulus in an appropriate way.

The nature of the Tbh dependent component was uncovered by the light induced preference experiment. This experiment shares features with intracranial self-stimulation (ICSS) used to measure the reinforcing properties of drugs [36]. In the ICSS an animal self administer electro shocks in specific brain regions by pressing a lever [37]. In our experiments, flies moved voluntarily to a region where octopaminergic neurons were activated by light in the presence of two identical odor choices. Difference in light intensity, flicker frequency or color can be excluded for this site preference, since the tested flies were visually impaired and the control animals did not show a preference for blue or yellow light. Conclusively, the choice to move towards the site with blue light was independent of olfactory and optical environmental cues, which supports a role for Tbh/octopamingeric neurons as a reinforcer. The vertebrate homologue of octopamine – noradrenalin – is also involved in mediating reward. Alteration of noradrenalinergic signaling by depletion of noradrenalin storage, inhibition of noradrenalin synthesis, or deletion of noradrenalin positive neurons blocks ICSS [38]. Supporting evidence that octopamine acts as a reinforcer comes from studies in *Drosophila* larvae. In an olfactory learning paradigm the reinforcer can be substituted for by the activation of tyraminergic/octopamingeric neurons [29]. Although the response to environmental factors like ethanol changes during development – larvae do not show any attraction to various ethanol concentrations [38], [39] whereas adult flies do [1] – the reinforcing properties of the octopamine/tyraminergic signaling appear to be maintained throughout development.

Is olfactory ethanol preference due to associative olfactory learning? Flies were reared in an ethanol free medium and in our assay flies were not previously exposed to ethanol. Furthermore, the only difference between the two odor sources was ethanol arguing that the observed behavior arises from an innate attraction rather association based learning. In addition, the light-induced activation of octopaminergic/tyraminergic neurons is independent of environmental cues. The reward center normally evaluates information of the external and internal environments processed via sensory systems. In turn the reward center influences memory formation and is involved in response selection. The ability to have a reinforcer is a prerequisite to perform a positive association of an odor with an unconditioned stimulus. Therefore, it might not be surprising that flies lacking octopamine fail to associate an odor and a sugar reward, since the reinforcer is missing [10]. Interestingly, the response level for light induced preference is comparable to a learning score in classical odor sugar memory, suggesting that the association of an odor with a reinforcer reflects the properties of the reinforcer/innate response.

Reward centers should communicate between rather widespread brain regions to form the interface between the external and internal environment on the input site and response selection and learning processes on the output site [40]. The reward center involved in olfactory preference should include brain centers involved in olfactory information processing and response selection, including the generation of locomotor output. The projections of the octopaminergic neurons involved in ethanol preference fulfill these criteria. Within the subset of neurons required for ethanol preference, neurons with synaptic varicosities and therefore putative output regions in areas involved in the olfactory pathway are found. For example, the ventral unpaired neuron VUMa2 projects to the mushroom bodies, the lateral horn, and the antennal lobes and shares morphological features with the honey bee VUMmx neuron, a neuron mediating rewarding properties [23], [16]. Furthermore, the paired ventral neuron VPM3 projects to the mushroom bodies and the central complex, a region involved in mediating locomotor performance [41]. The AL2i2 neuron of the antennal lobes clusters putatively receives input from the protocerebrum and has varicosities in the region of the protocerebral bridge, a part of the central complex. Interestingly, putative input regions as visualized by spiny ramifications of the octopaminergic neurons are found in the inferior and superior posterior slope [23] and which are good candidate regions to gather internal information.

In summary, the function of Tbh in olfactory preference is found at the interface between sensory information and response selection and acts as a reward center. How the octopamine/Tbh dependent reinforcer works – whether this happens through shifts in attention or a general incentive motivation or an internal drive – needs to be further determined.

## Materials and Methods

### Strains

Flies were raised on ethanol free standard cornmeal/molasses/yeast/agar medium on a 12/12 h light and dark cycle at 25°C with 60% humidity. Flies for temperature shift experiments were kept at 18°C with 60% humidity on a comparable light and dark regime.

The following lines were used *w^1118^,Orco^1^* and *w^1118^*,*Orco^2^* [5]. *w^1118^,Tbh^nM18^* and the respective *w^1118^* background [21]. *w^+^,Tbh^nM18^* and the respective *w^+^* background. *w^1118^*,*Tdc2*-GAL4 [18]. *w^1118^*;*Feb15*-GAL4 [42]. y^1^,w^*^;P{UAS-mCD8::GFP.L}LL5 [25]. *Tub-*GAL80^ts^ [28]. *w^1118^*,UAS-*Kir2.1* [43]. *w^1118^*;*Cha*-GAL80 [26]. *w^1118^*,*TH*-GAL4 [44]. *norpA^1^*;UAS-ChR2. UAS-*ChR2* [45]. Flies carrying transposable elements were backcrossed for at least five generations to the *w^1118^* background maintained in the laboratory before use.

### Generation of Transgenic Strains

The *UAS*-*Tbh* transgene was generated by cloning a 3 kb KpnI/NotI fragment from the pBS-*Tbh* cDNA plasmid (kindly provided by Maria Monastirioti) downstream of the UAS sequence contained in the pUAST transformation vector. A 6.2 kb promoter fragment of the *Tbh* gene ranging from −5750 to +360 was amplified by using the primers 5′-GCTGCTCGACCAATTTTAACG-3′ and 5′-CCAAGATGCTAACGGTAATGG-3′. The fragment was cloned into the pCRII vector (LifeTechnologies) and from there into the p221-4 GAL4 vector using the restriction sites KpnI and XbaI. Both constructs were transformed into *w^1118^* flies to generate transgenic lines.

### Preference Assay

To determine odor preference a two vial choice assay was used offering food odor with 5% ethanol and without ethanol if not otherwise indicated (Figure 1a). For behavioral experiments populations of 80 male flies were collected using CO_2_ for anesthesia. After 2 days of recovery at 25°C the flies (3 to 5 days old) were used for behavioral experiments. The experiments were performed as described in Ogueta *et al.*, 2010.

### Statistics

Bars labeled with **a** are significantly different from random choice (One-sample sign test). Because the data were normally distributed for comparison of two experimental groups the Student’s *t*-test was used. When more than two experimental groups were tested, ANOVA post Tukey-Kramer was used. Errors are indicated as standard error of the mean (SEM).

### Temperature Shift Experiments

*Tdc2*-GAL4*/*UAS-*Kir2.2*;*Tub-*GAL80^ts^ and *w^1118^*;UAS*-Kir2.2*;*Tub-*GAL80^ts^ received a heat shock for 16 h at 33°C. After the heat shock they were left to recover at 18°C for 9 h before they were tested in the two choice assay.

### Optogenetic Experiments

Flies expressing UAS-*ChR2*;UAS-*ChR2* were raised on standard media containing 150 µl of either ethanol for control flies or 150 mM all-trans retinal (Sigma, Germany) dissolved in ethanol. After hatching 80 male flies were collected with CO_2_ and then fed on small vials with about 30 ml fly media, also containing ethanol or all-trans retinal in ethanol for 2 days. The 3 to 5 days old flies were then tested for juice only in a dark apparatus on a cold light plate. In the lid, the apparatus was illuminated by two different light sources. For blue light illumination a LED (465–485 nm; Cree, Germany) and for yellow light illumination a warm white LED (Cree, XLAMP, XR_E LED with 2,600 k–3,700K CCT) with a 510 nm yellow filter (HEBO, Aalen, Germany) were used. The following light sequence was repeated over a period of 16 h for both LEDs: 50 Hz for 2 s, followed by 16 s with 5 Hz and 2 s constant light. The intensities of the LEDs were standardized to 200 000 lx.

### Immunohistochemistry and Imaging of Whole Mount Brains

After CO_2_ anesthesia 3 to10 days old male flies were dissected in ice cold *Drosophila* Ringer, transferred to PBS (pH 7.4) and fixed with 3.7% formaldehyde for 30 min at room temperature, washed with PBS containing 0.5% Triton X-100 (PBT) and blocked for 1 h in 5% fetal calf serum in PBT. Primary antibodies were used in the appropriate dilutions prepared in 5% fetal calf serum in PBT and incubated overnight at 4°C. The next day the tissue was washed at least three times for 20 minutes and incubated with the secondary antibodies in the appropriate dilutions for 2 h at room temperature or overnight at 4°C. After washing whole mounts were placed in 50% glycerol for 30 minutes and then mounted in Vectashield.

The following antibodies were used: Mouse anti-GFP (LifeTechnologies, 1∶50) or chicken anti-GFP (LifeTechnologies, 1∶1000) and rabbit anti-TbH (generously provided by Yi Rao, 1∶500). AlexaFluor 488 goat anti-mouse IgG (LifeTechnologies, 1∶200), AlexaFluor 488 goat anti-chicken IgG (LifeTechnologies, 1∶500) and Cy3 goat anti-rabbit IgG (Jackson Immunoresearch, 1∶200) or AlexaFluor 546 goat anti-rabbit IgG (LifeTechnologies, 1∶500).

Confocal imaging was performed with a Zeiss LSM 510 laser scanning microscope. Data analysis of confocal stacks were done with ImageJ (http://rsbweb.nih.gov/ij/).

## Supporting Information

Figure S1 Restricting *Tdc2*-GAL4 by *Cha*-GAL80 reduces neurons positive for TbH and GFP.
(TIF)

Figure S2 *Feb15*-GAL4 drives expression the ventral neurons.
(TIF)

Figure S3 *6.2*-Tbh-GAL4 drives expression in the VUMa4 neuron.
(TIF)

Figure S4 The expression of Tdc2-GAL4 line does not match all TbH positive neurons in the thorax.
(TIF)

Figure S5 The Tdc2-GAL4; Cha-GAL80 drives expression in a subset of TbH positive neurons.
(TIF)

Figure S6 *NP-7088*-GAL4 dependent expression of TbH restores preference of *Tbh^nm18^* mutants.
(TIF)

Table S1 Concentration independent loss of preference in *Tbh^nm18^*.
(TIF)

Table S2 TbH expression in the adult head.
(TIF)

Table S3 Expression patterns of GAL4 driver lines in the thorax in comparison to TbH.
(TIF)
