# Supplementary figures and images for: Neuronal Basis of Innate Olfactory Attraction to Ethanol in Drosophila
Source: PLoS One. 2012 Dec 20;7(12):e52007. doi: 10.1371/journal.pone.0052007 (PMC3527413; doi:10.1371/journal.pone.0052007)

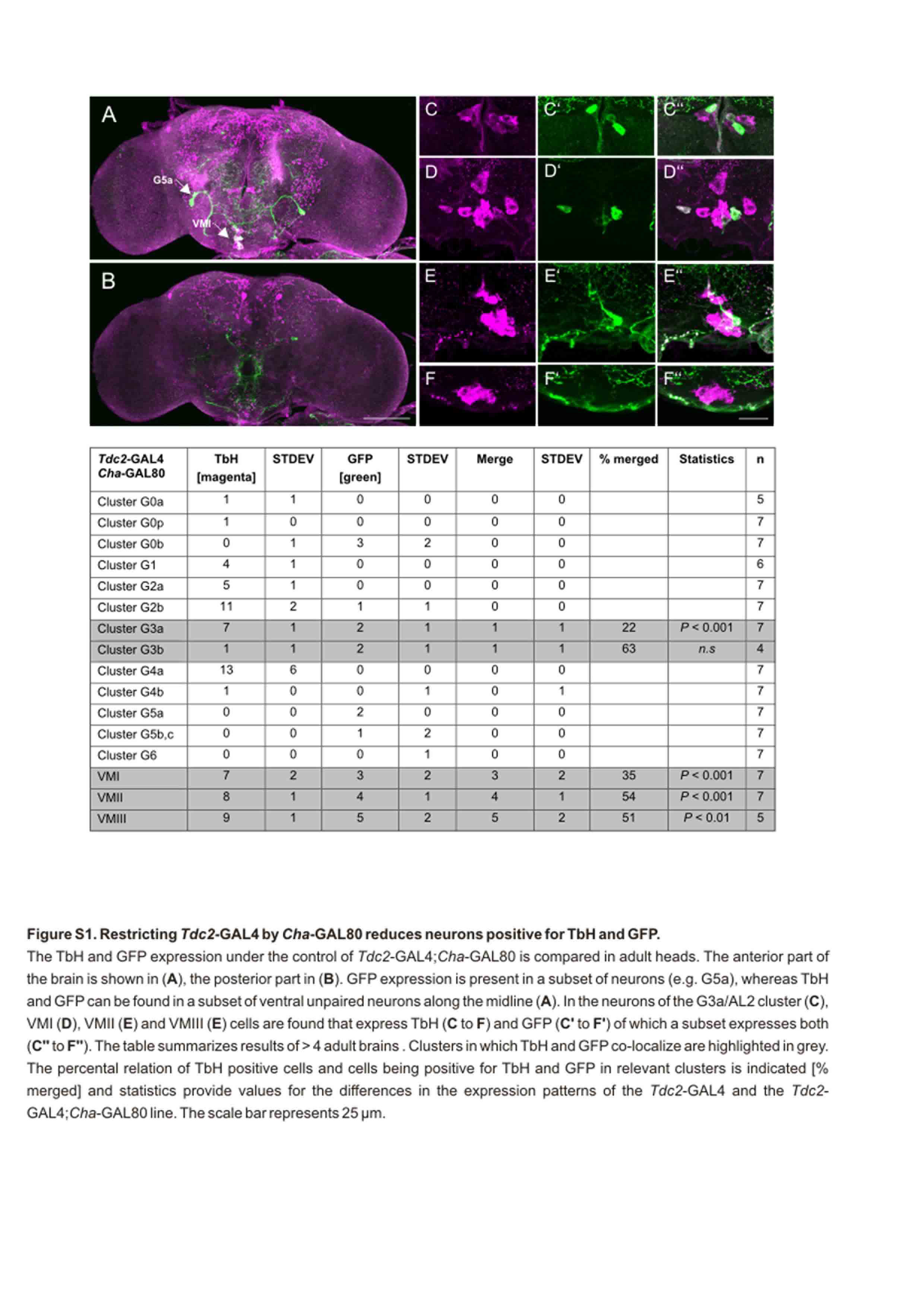

Supplement: Figure S1 — Restricting Tdc2-GAL4 by Cha-GAL80 reduces neurons positive for TbH and GFP. (TIF) [file pone.0052007.s001.tif]

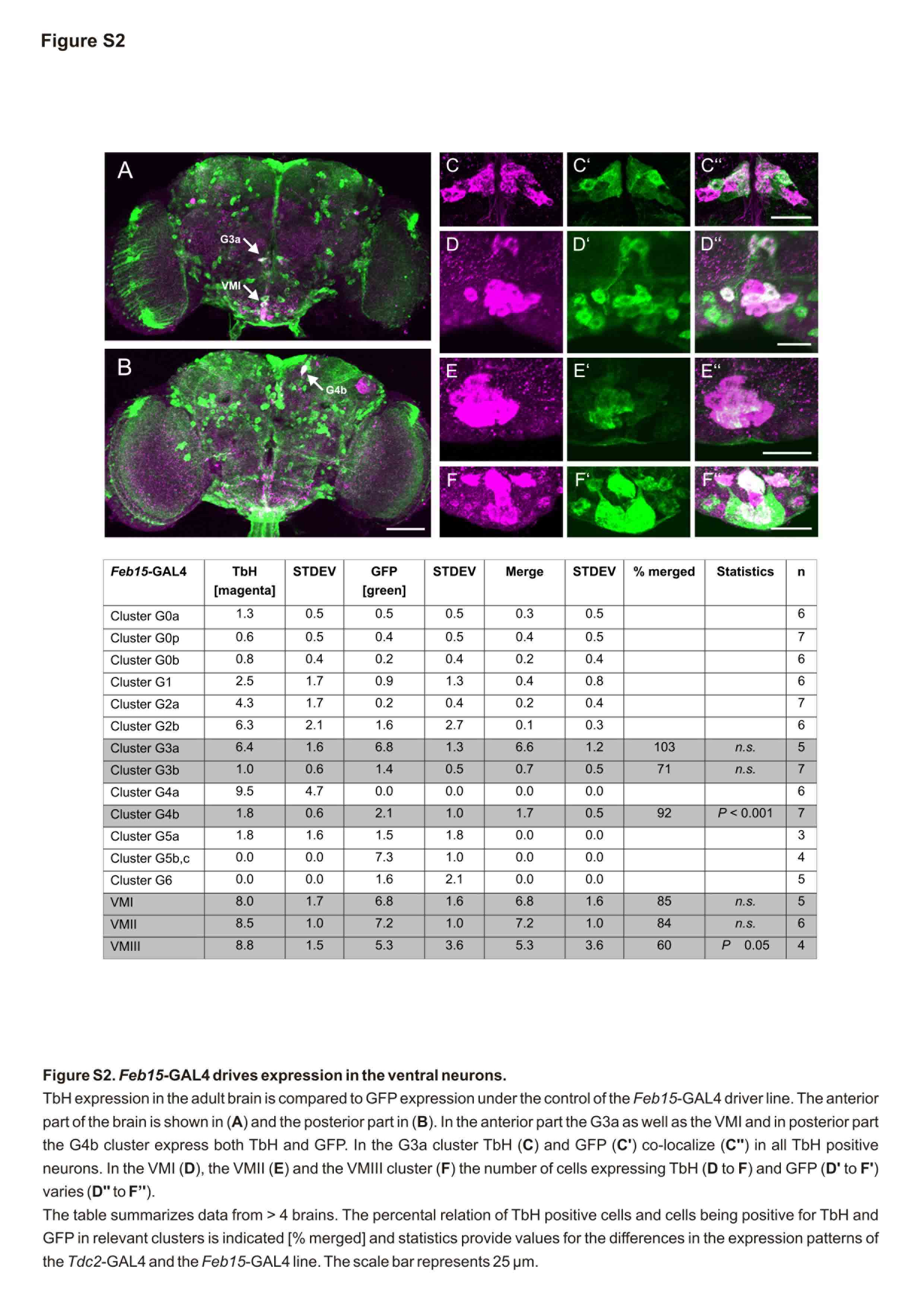

Supplement: Figure S2 — Feb15-GAL4 drives expression the ventral neurons. (TIF) [file pone.0052007.s002.tif]

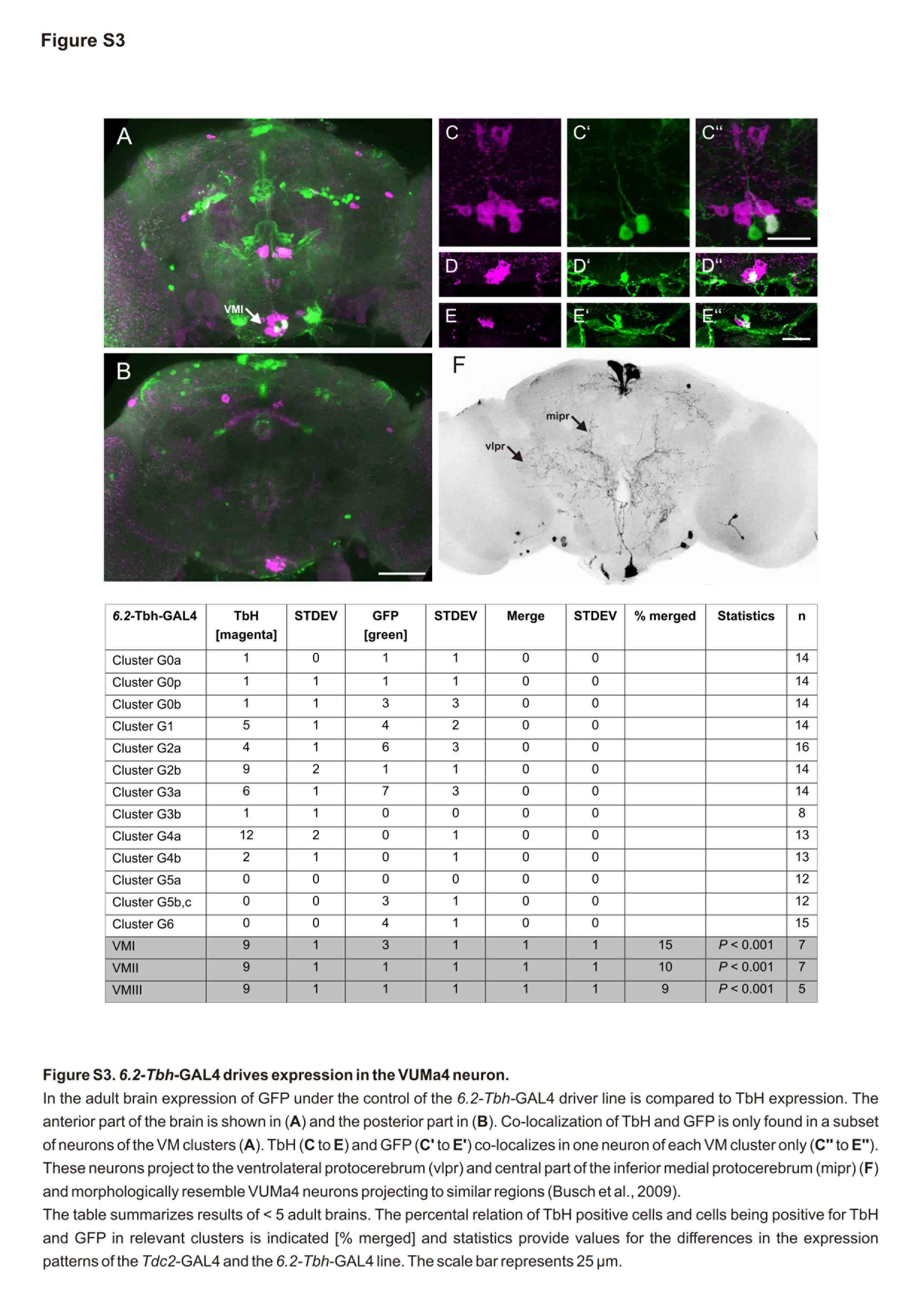

Supplement: Figure S3 — 6.2-Tbh-GAL4 drives expression in the VUMa4 neuron. (TIF) [file pone.0052007.s003.tif]

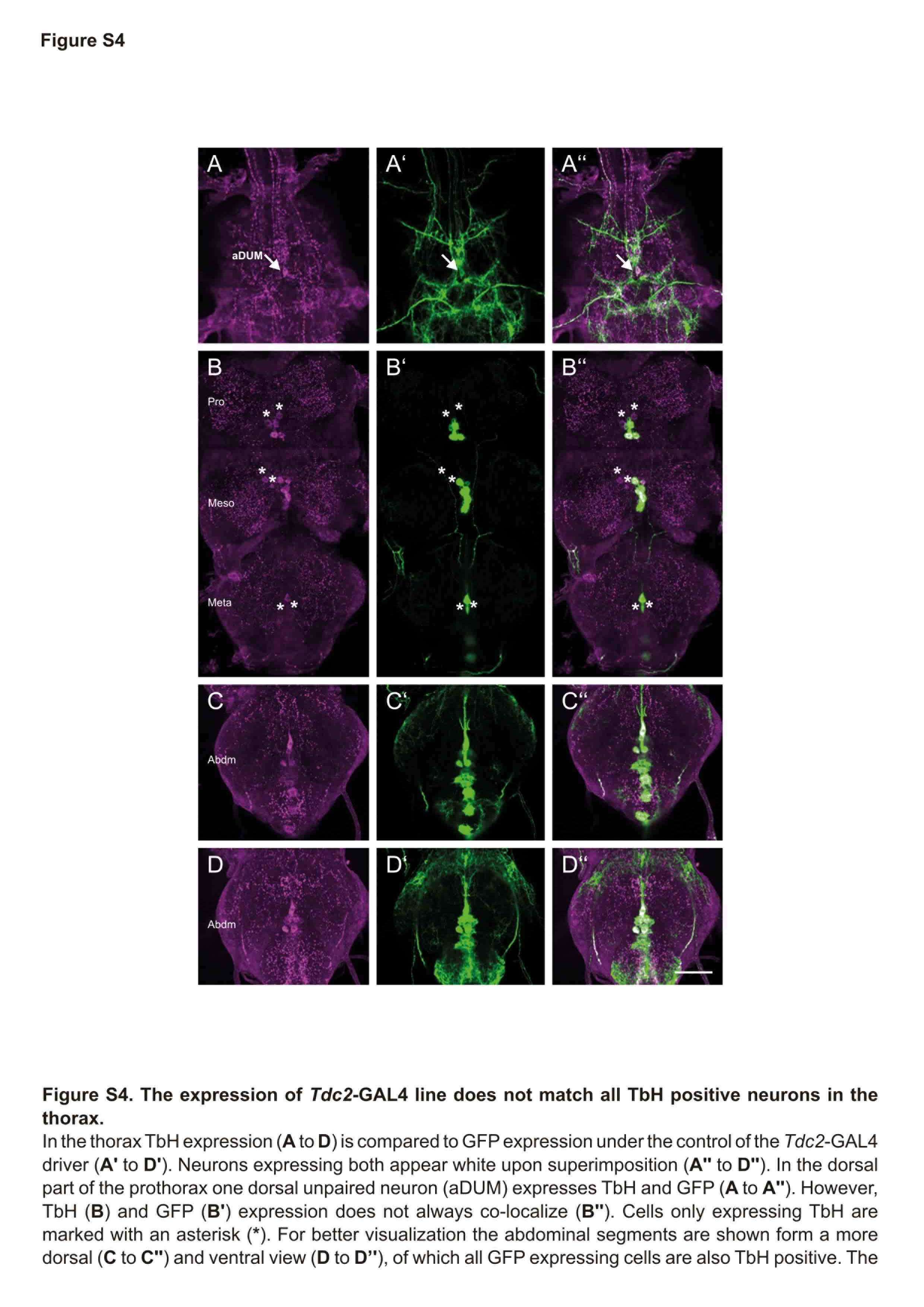

Supplement: Figure S4 — The expression of Tdc2-GAL4 line does not match all TbH positive neurons in the thorax. (TIF) [file pone.0052007.s004.tif]

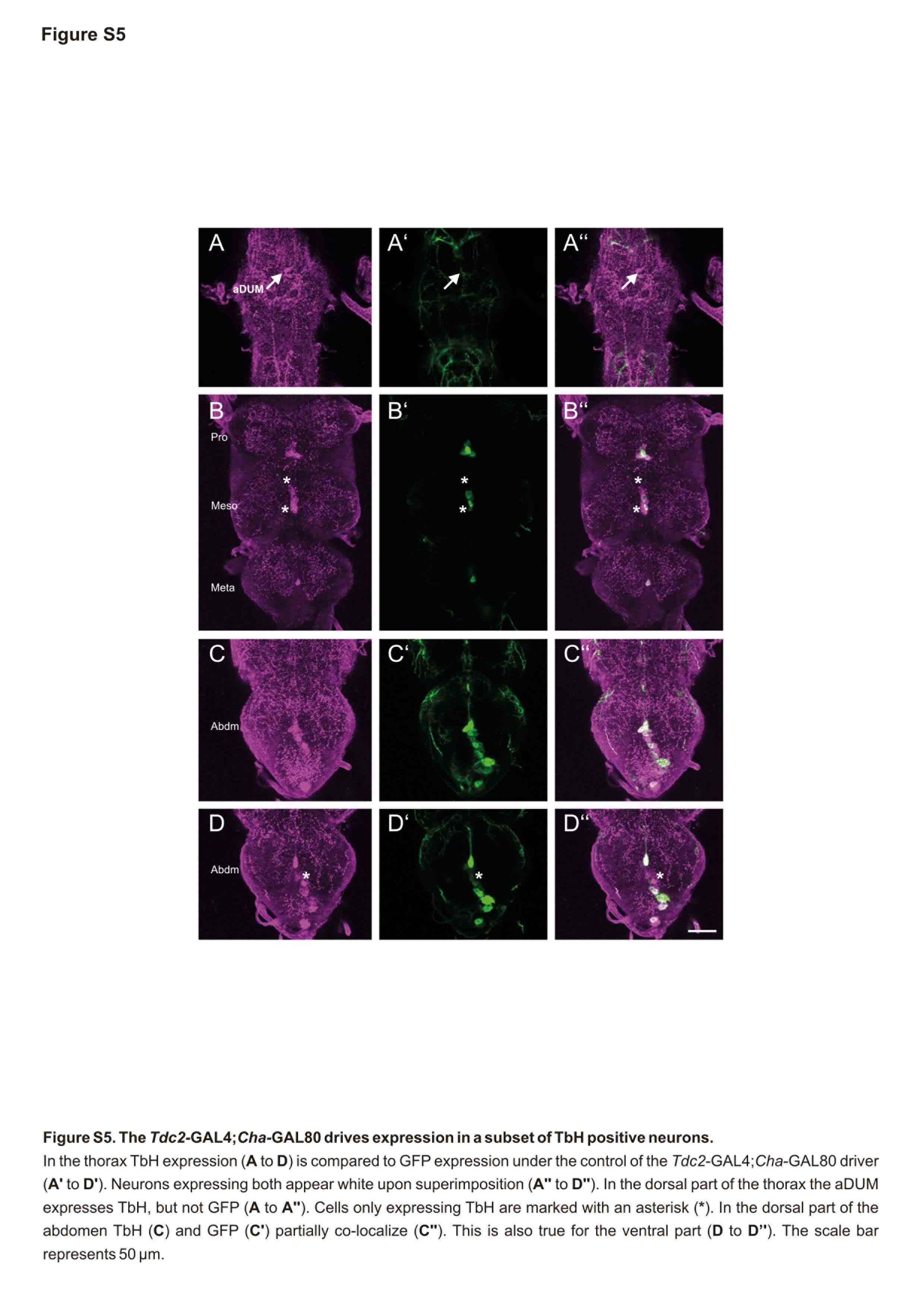

Supplement: Figure S5 — The Tdc2-GAL4; Cha-GAL80 drives expression in a subset of TbH positive neurons. (TIF) [file pone.0052007.s005.tif]

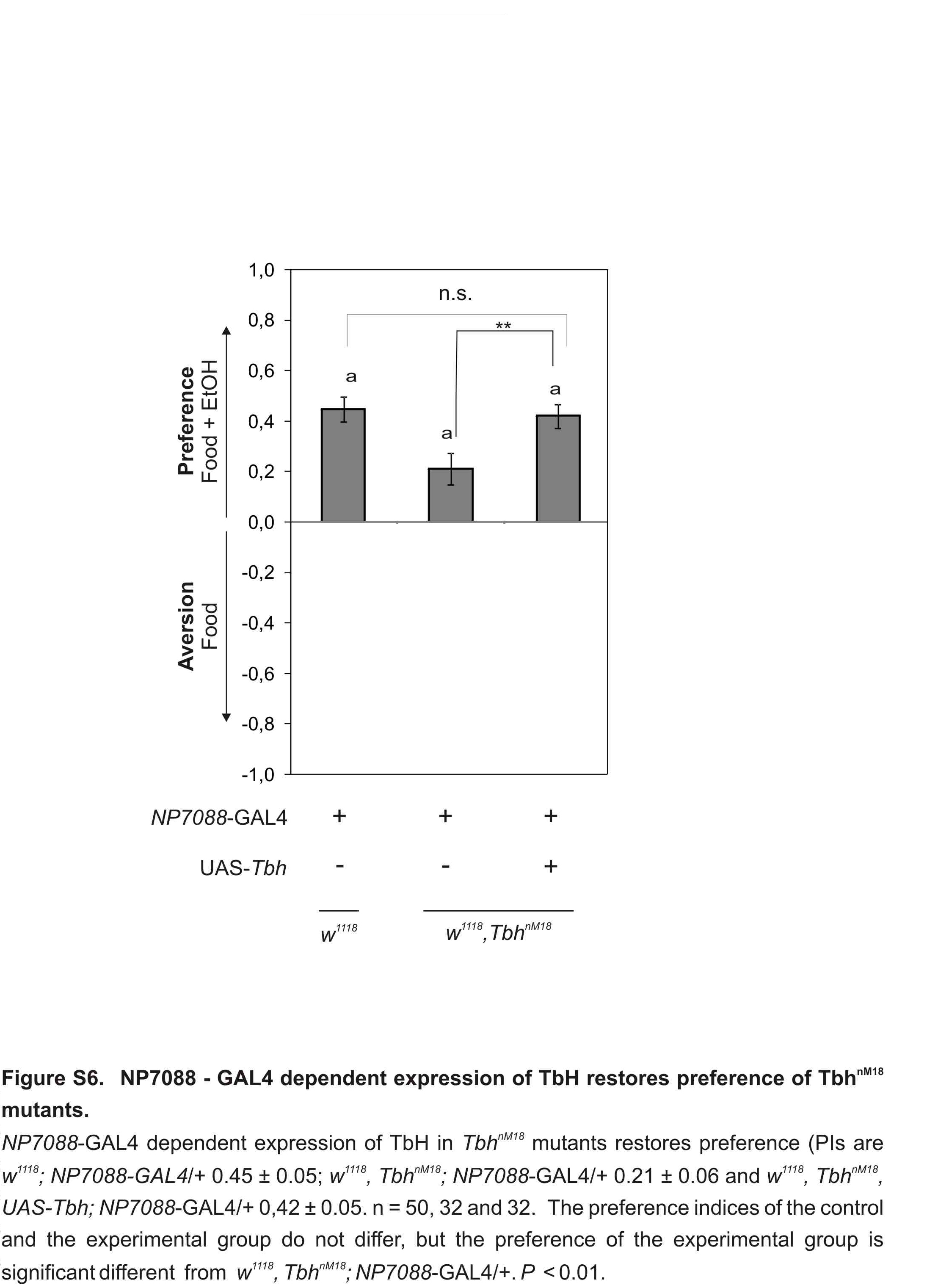

Supplement: Figure S6 — NP-7088-GAL4 dependent expression of TbH restores preference of Tbhnm18 mutants. (TIF) [file pone.0052007.s006.tif]

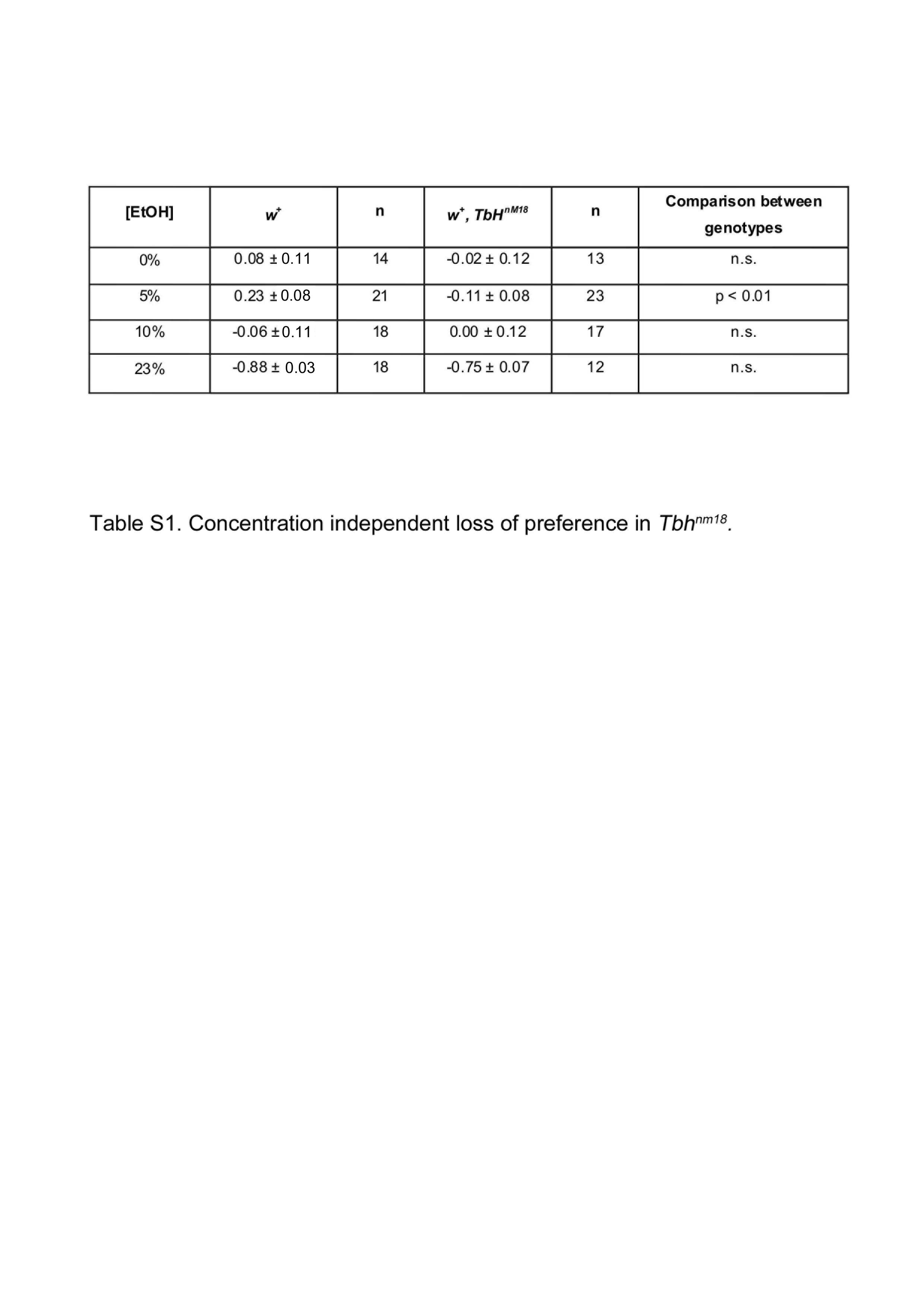

Supplement: Table S1 — Concentration independent loss of preference in Tbhnm18. (TIF) [file pone.0052007.s007.tif]

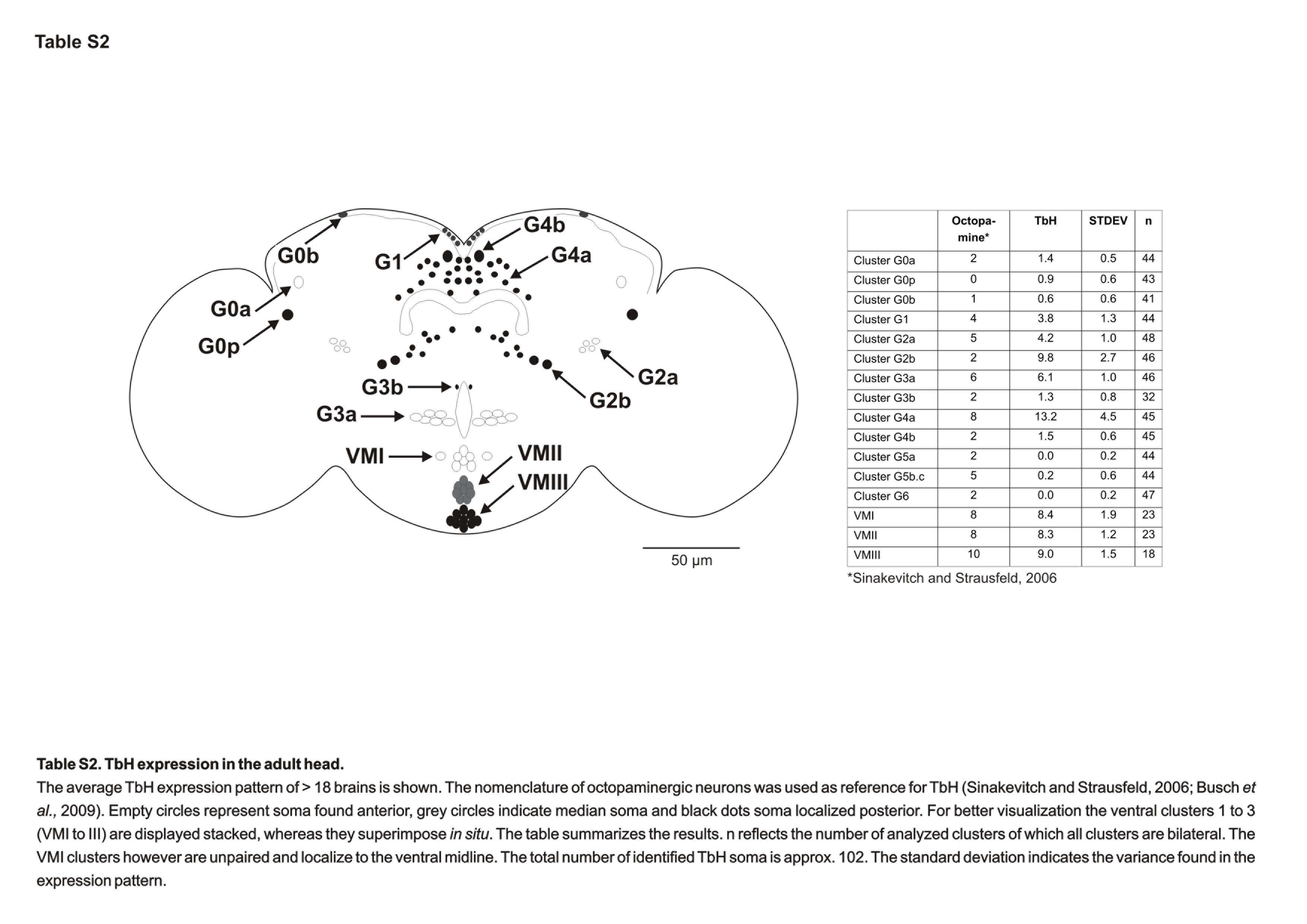

Supplement: Table S2 — TbH expression in the adult head. (TIF) [file pone.0052007.s008.tif]

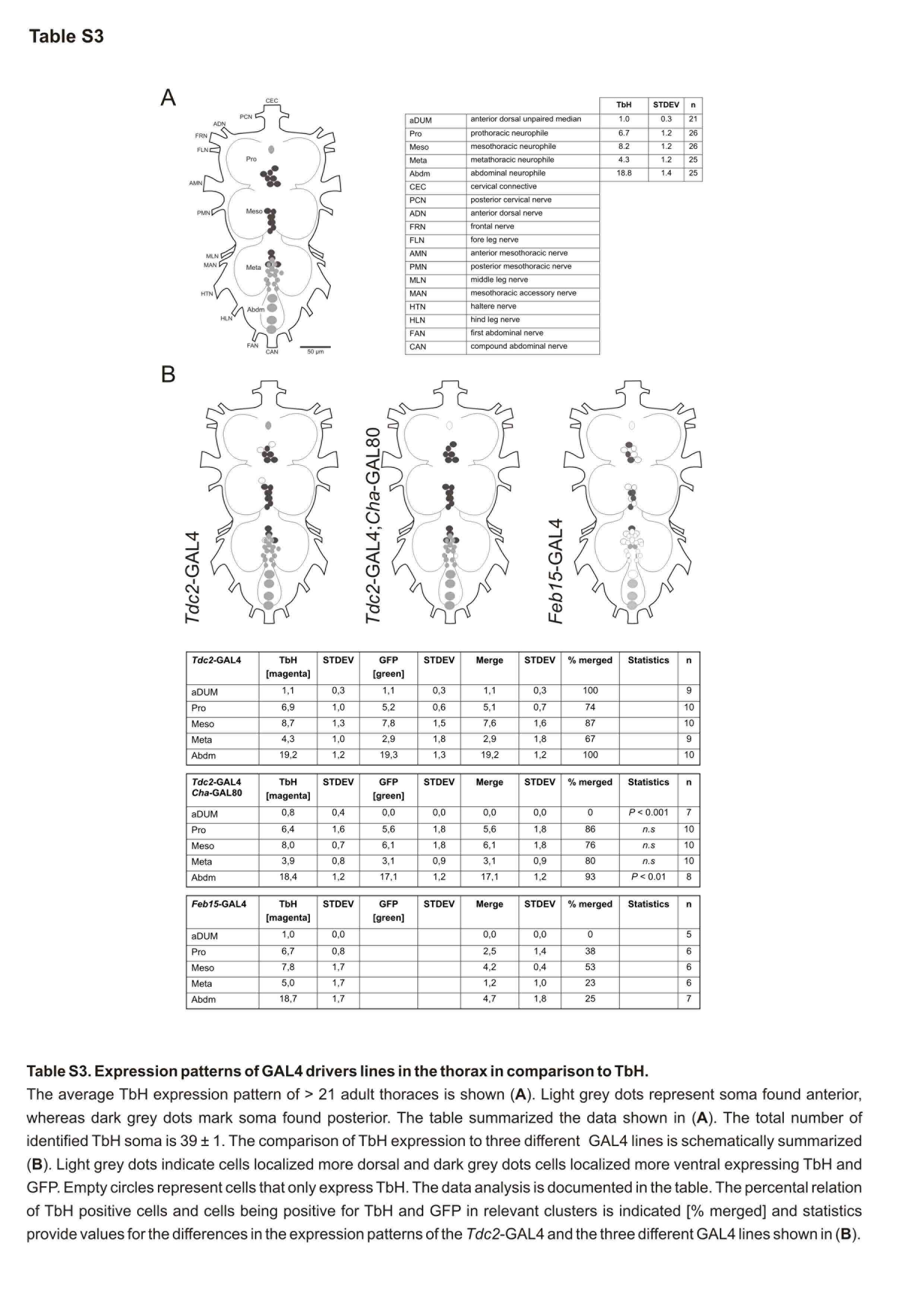

Supplement: Table S3 — Expression patterns of GAL4 driver lines in the thorax in comparison to TbH. (TIF) [file pone.0052007.s009.tif]
